# Sex Steroid Hormones as a Balancing Factor in Oral Host Microbiome Interactions

**DOI:** 10.3389/fcimb.2021.714229

**Published:** 2021-09-29

**Authors:** Pilar Cornejo Ulloa, Bastiaan P. Krom, Monique H. van der Veen

**Affiliations:** Department of Preventive Dentistry, Academic Centre for Dentistry Amsterdam (ACTA), University of Amsterdam and VU University Amsterdam, Amsterdam, Netherlands

**Keywords:** sex steroid hormones, host-microbiome interactions, microbial endocrinology, oral microbiome, oral bacteria and fungi

## Abstract

Sex steroid hormones (SSH) are cholesterol-derived molecules. They are secreted into saliva and enter the oral cavity, triggering physiological responses from oral tissues, with possible clinical implications, such as gingival inflammation and bleeding. SSH and hormonal changes affect not only oral host cells but also oral microorganisms.

Historically, most research has focused on the effect of hormonal changes on specific bacteria and yeasts. Recently a broader effect of SSH on oral microorganisms was suggested. In order to assess the role of SSH in host-microbe interactions in the oral cavity, this review focuses on how and up to what extent SSH can influence the composition and behavior of the oral microbiome. The available literature was reviewed and a comprehensive hypothesis about the role of SSH in host-microbiome interactions is presented. The limited research available indicates that SSH may influence the balance between the host and its microbes in the oral cavity.

## 1 Introduction

**Figure 1:**
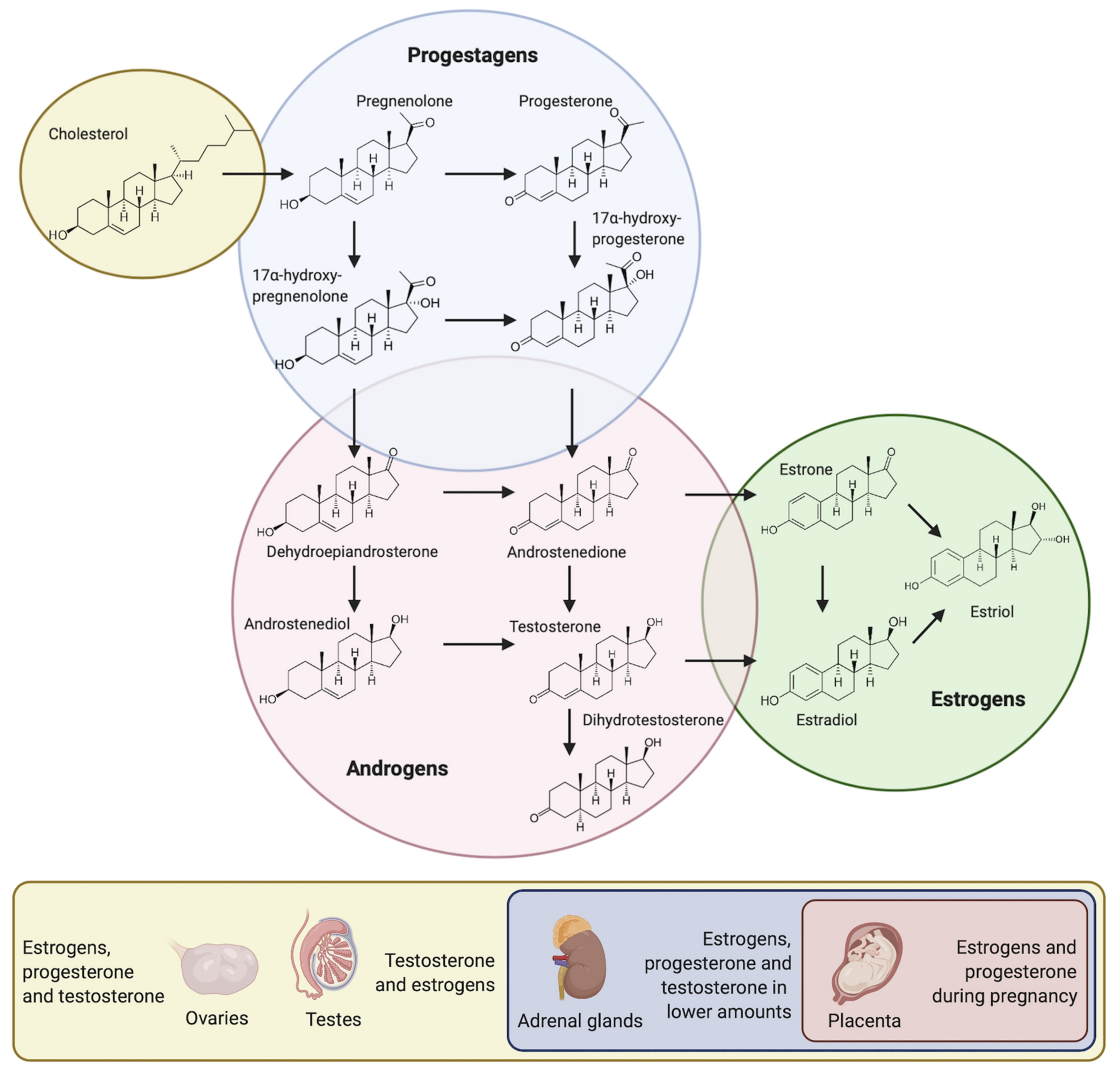
Pathways for the synthesis of SSH and secretory organs in the human body.

Sex steroid hormones (SSH) are regulatory molecules synthesized or converted by several different living organisms ranging from mammals to plants, and from parasites to bacteria (Winter and Bokkenheuser, 1987; Kristan and Rizner, 2012; Romano et al., 2015; Chiang et al., 2019; Tarkowská, 2019). In mammals, these molecules derive from cholesterol and are secreted by the testes, ovaries, adrenal cortex and – during pregnancy – by the placenta (**Figure 1**). After being secreted, they are released into the bloodstream to reach different target tissues, where they induce various physiological functions (Chan and O'Malley, 1976; Hu et al., 2010). Interestingly, even though SSH are structurally similar, they induce very different responses in target tissues (**Figure 1**). The oral cavity is not exempt from the effect of these molecules. In fact, SSH can diffuse into saliva through capillaries and salivary ducts (Vining et al., 1983). Consequently, they are capable of reaching intra-oral target tissues such as the gingiva and the periodontium, where they induce a response (Markou et al., 2009; Mariotti and Mawhinney, 2013). Likewise, microorganisms present in the oral cavity are exposed to SSH and specific oral bacteria and yeast have been shown to be affected by them (Kornman and Loesche, 1982; Powell et al., 1984).

During pregnancy, the surge in hormonal levels triggers responses by oral tissues, with 30-100% of women undergoing gingival changes such as inflammation and increased bleeding (Mealey and Moritz, 2003). Similar changes have also been observed during physiological and artificially induced hormonal shift periods, as well as an effect on oral bacteria (Ozcelik et al., 2006; Kumar, 2013; Brusca et al., 2014).

Researchers have tried to explain why oral clinical changes take place, which mechanisms are involved, and the possible implications of both for the host. It has been proposed that SSH are capable of affecting periodontal tissues and their surrounding environment through four different mechanisms: (1) by locally influencing proliferation of fibroblasts and epithelial cells; (2) by increasing vascular permeability; (3) by elevating levels of immune cells in the periodontium; and (4) by inducing changes in certain oral microorganisms (Mariotti and Mawhinney, 2013). However, despite the available evidence, it is not yet known how these mechanisms are connected and what the exact influence of SSH is on periodontal tissues and the oral microbiota.

Studies in females and males on the effects of SSH on oral bacteria and fungi have been inconclusive. Moreover, the study of the oral microbiome, understood as the microorganisms present in the oral cavity, their genetic information and the environment in which they interact (Cho and Blaser, 2012) has gained interest (Cornejo Ulloa et al., 2019) and represents a gap in our knowledge that needs to be addressed. Developments in the field of microbial endocrinology will especially help to better understand periodontal health and disease. In addition, current omics-based techniques open up vast possibilities to study the effects of SSH on not only single-species but also the oral microbial composition in depth.

The purpose of this review was to assess the role of SSH in host-microbe interactions in the oral cavity, and the extent to which SSH can influence the composition and behavior of the oral microbiome. Our review of *in vitro* and clinical studies provided the basis for a hypothesis about host-microbiome interactions mediated by SSH. Possible implications for the host’s oral health and future perspectives are discussed.

## 2 *In Vitro* Studies

Just like other microorganisms, some oral bacteria are capable of transforming or degrading SSH (Kornman and Loesche, 1982; Clark and Soory, 2007; Donova and Egorova, 2012; Holert et al., 2018; Horinouchi et al., 2019). This has been tested *in vitro* using estradiol, progesterone and testosterone on different oral microorganisms, especially periodontal bacteria. The specific focus on periodontal bacteria derives from earlier studies showing that SSH can compensate for the absence of vitamin K (Kornman and Loesche, 1982). Vitamin K is produced *in vivo* by certain microorganisms and further used as an electron carrier during the anaerobic reduction of fumarate to succinate by periodontal bacteria (Glick et al., 1959; Thauer et al., 1977; Marcotte and Lavoie, 1998; Hojo et al., 2007).

Several studies have investigated the response of different oral bacteria and the fungus *Candida* to the presence of SSH and other similar compounds (Ojanotko-Harri et al., 1991; Soory, 1995; Clark and Soory, 2005; Clark and Soory, 2007; Fteita et al., 2014) and have also suggested that the exposure of certain microorganisms to SSH can modify bacterial metabolism and the growth and expression of virulence factors (Garcia-Gomez et al., 2013), and have effects on the host (Vom Steeg and Klein, 2017).

Unfortunately, most *in vitro* studies have used single-species models (Kornman and Loesche, 1982; Clark and Soory, 2005; Yokoyama et al., 2005), while fewer studies have worked with polymicrobial models (Soory, 1995; Soory and Ahmad, 1997) to mimic a more realistic oral environment. While this has simplified the understanding of single-species behavior, it has also created reductionistic inferences – which, as a matter of fact, do not necessarily apply when looking at the bigger picture: the oral microbiome and its interplay with the host.

To date, *in vitro* research has sought to establish the direct and indirect effects of certain oral species, i.e. (1) the interaction between oral microorganisms and SSH; and (2) the response of host oral cells to the presence of both hormone metabolizing microorganisms and SSH. These direct and indirect influences of SSH on oral microbiota are discussed below.

### 2.1 The Interaction Between Oral Microorganisms and Sex Steroid Hormones: Who Are They and How Do They React?

Several oral bacteria and *Candida* species have been reported to respond to the presence of SSH. For the purpose of this review, SSH-responsive microorganisms have been classified into four groups based on their putative role in oral health and disease and in line with the classification used in the majority of the reviewed literature. These are: (1) periodontal-disease-associated bacteria; (2) caries-associated bacteria; (3) oral-health-associated bacteria; and (4) oral fungi. Their characteristics and known response to SSH are summarized in **Table 1**.

**Table 1:** Studies that have tested the response of different oral microbes to SSH *in vitro*.

	Species	Enz. activity	Gram	Respiration	Capsule	Responsive to Estrogen?	How?	Type of culture	Responsive to Progestagens?	How?	Type of culture	Responsive to Androgens?	How?	Type of culture
**Periodontitis associated bacteria**	***Aggregatibacter actinomyce-temcomitans***	Pr	–	FA	–	Unk	N/A	N/A	Unk	N/A	N/A	Yes	Testosterone was metabolized into DHT and 4-androstenedione (Soory, 1995)	P
	***Bacillus cereus***	Pr	+	FA	–	Yes	Metabolism of 17β estradiol (Ojanotko-Harri et al., 1990)	P	Yes	Detection of 5α-steroid hydrogenase, 3β- 17β- and 20α-hydroxysteroid dehydrogenase and steroid hydroxylase indicating metabolism of progesterone (Ojanotko-Harri et al., 1991)	P	Yes	Detection of 5α-steroid hydrogenase, 3β- 17β- and 20α-hydroxysteroid dehydrogenase and steroid hydroxylase indicating metabolism of testosterone (Ojanotko-Harri et al., 1991)	P
	***Campylobacter rectus***	Pr	–	FA	–	Yes	Increased growth when estradiol was added to the culture medium (Yokoyama et al., 2005)	P	Yes	Increased growth when progesterone was added to the culture medium (Yokoyama et al., 2005)	P	Unk	N/A	N/A
	***Prevotella aurantiaca***	Pr	–	A	–	Yes	Estradiol regulates DPPIV enzyme activity (Fteita et al., 2015)	B	Unk	N/A	N/A	Unk	N/A	N/A
	***Prevotella intermedia***	Pr	–	A	–	Yes	Substitution of vitamin K as growth factor (Kornman and Loesche, 1982)	P	Yes	Substitution of vitamin K as growth factor (Kornman and Loesche, 1982)	P	Yes	Testosterone was metabolized into DHT and 4-androstenedione (Soory, 1995)	P
							Increased growth when estradiol present in culture medium (Fteita et al., 2014)	B,P						
							Estradiol regulates planktonic growth, coaggregation, polysaccharide production and biofilm formation differently for different strains (Fteita et al., 2014)	B,P						
	***Prevotella intermedia***	Pr	–	A	–	Yes	Estradiol regulates DPPIV enzyme activity (Fteita et al., 2015)	B		Increased growth when progesterone was added to the culture medium (Fteita et al., 2014)	B,P	Yes	See previous page	P
	***Prevotella melaninogenica***	Pr	–	A	–	Yes	Substitution of vitamin K as growth factor (Kornman and Loesche, 1982)	P	Yes	Substitution of vitamin K as growth factor (Kornman and Loesche, 1982)	P	Unk	N/A	N/A
	***Prevotella nigrescens***	Pr	–	A	–	Yes	Estradiol regulates planktonic growth, coaggregation, polysaccharide production, and biofilm formation differently for different strains (Fteita et al., 2014)	B,P	Unk	N/A	N/A	Unk	N/A	N/A
							Estradiol regulates DPPIV enzyme activity (Fteita et al., 2015)	B						
	***Prevotella pallens***	Pr	–	A	–	Yes	Estradiol regulates DPPIV enzyme activity (Fteita et al., 2015)	B	Unk	N/A	N/A	Unk	N/A	N/A
							Estradiol regulates planktonic growth, coaggregation, polysaccharide production, and biofilm formation differently for different strains (Fteita et al., 2014)	B,P						
	***Porphyromonas gingivalis***	H Pr	–	A	+	Yes	In the presence of fumarate, increased uptake of estrogen and detection of succinate (Kornman and Loesche, 1982)	P	Yes	In the presence of fumarate, increased uptake of Progesterone and detection of succinate (Kornman and Loesche, 1982)	P	Yes	Testosterone was metabolized into DHT and 4-androstenedione (Soory, 1995)	P
	***Treponema denticola***	H Pr	–	A	–	Unk	N/A	N/A	Yes	Metabolism of progesterone (Clark and Soory, 2005)	P	Yes	Metabolism of testosterone and 4-androstenedione (Clark and Soory, 2005)	P
										T. denticola ATCC 35520 has 5a-R activity with a specificity for progesterone (Clark and Soory, 2007)	P		T. denticola has 5a-R activity with a specificity for 4-androstenedione, testosterone in a lower degree than for progesterone (Clark and Soory, 2007)	P
										Progesterone is capable of modulating the growth of T. denticola (Clark and Soory, 2006)	P		Testosterone and 4-androstenedione are capable of modulating the growth of T. denticola (Clark and Soory, 2006)	P
**Caries associated bacteria**	***Streptococcus mutans***	S	+	FA	+	Yes	Metabolism of 17β estradiol (Ojanotko-Harri et al., 1990)	P	Unk	N/A	N/A	Unk	N/A	N/A
**Health associated bacteria**	***Streptococcus sanguinis***	S	+	FA	+	Yes	Metabolism of 17β estradiol (Ojanotko-Harri et al., 1990)	P	Unk	N/A	N/A	Unk	N/A	N/A
**Oral fungi**	***Candida albicans***	Pr S	+	FA	–	Yes	Metabolism of 17β estradiol (Ojanotko-Harri et al., 1991)	P	Yes	Progesterone stimulates germination (Kinsman et al., 1988)	P	Unk	N/A	N/A
							Binding to 17β estradiol (Powell et al., 1984; Skowronski and Feldman, 1989; Gujjar et al., 1997)	P						
							Stimulates growth (Gujjar et al., 1997)	P						
							Stimulates germination (Kinsman et al., 1988; White and Larsen, 1997)	P						
							Up-regulation of intracellular *hsp90* and increased expression of *CDR1* (Zhang et al., 2000)	P						
							17β-estradiol and Ethynyl estradiol promoted increase in cells forming germ tubes and its length. Increased expression of *CDR1* and of *CDR2* (Cheng et al., 2006)	P						
							Induces expression of *CDR1* (Krishnamurthy et al., 1998; Larsen et al., 2006)	P		Progesterone induces expression of *CDR1* (Larsen et al., 2006)	P			
										Progesterone reduces capacity to form biofilms, colonize and invade epithelial cells. It also decreases the expression of *BCR1* and *HWP1* (Alves et al., 2014)	B			
	***Candida glabrata***	Pr S	+	FA	–	Yes	Binding to 17β estradiol (Powell et al., 1984)	P	Unk	N/A	N/A	Unk	N/A	N/A

H, hydrolytic; Pr, proteolytic; S, saccharolytic; A, aerobic; FA, facultative anaerobic; Green, effects on growth; Yellow, effects on metabolism; Orange, effects on virulence; P, planktonic; B, biofilm.

#### 2.1.1 Periodontal Disease Associated Bacteria

In the early 1980s, Kornman and Loesche conducted the first study which showed that, in the absence of vitamin K, two periodontal bacteria – *Bacteroides melaninogenicus* subsp. *intermedius* (now *Prevotella intermedia*), and *Bacteroides melaninogenicus* subsp. *melaninogenicus* (now *Prevotella melaninogenica*) – used estradiol and progesterone as growth factors in the absence of vitamin K (Kornman and Loesche, 1982). They also showed that *Bacteroides gingivalis* (now *Porphyromonas gingivalis*) and *P. intermedia* were able to nearly quintuplicate their SSH uptake when fumarate was added to the medium, significantly enhancing their metabolism.

Later studies have confirmed these observations and identified other effects of estradiol on bacteria from the *Prevotella intermedia* group. Estradiol positively increases protein levels and formation of polysaccharides in biofilms as well as enhancing its coaggregation with *Fusobacterium nucleatum*. This could have an influence in biofilm formation (Fteita et al., 2014). Moreover, *Prevotella intermedia* group bacteria express enzymes that act as virulence factors. One of these enzymes is the protease dipeptidyl peptidase IV (DPPIV) which is involved in the destruction of periodontal tissue. The activity of this protease is increased by the presence of estradiol, which thus enhances its virulence (Fteita et al., 2015).

The ability of certain periodontal pathogens to metabolize testosterone has also been tested. Subgingival plaque samples as well as single-species cultures of *Aggregatibacter actinomycetemcomitans, Prevotella intermedia and Porphyromonas gingivalis* were grown in the presence of testosterone. Both subgingival plaque samples and single-species cultures were able to convert testosterone into dihydrotestosterone (DHT) or 4-androstenedione (Soory, 1995) (**Figure 1**). Two years later, a similar experiment was conducted to assess the effect of these bacterial species alone, in pairs or together in the conversion of 4-androstenedione to testosterone or DHT. It was observed that *A. actinomycetemcomitans, P. intermedia* and *P. gingivalis* were able to convert 4-androstenedione into testosterone and DHT. Additionally, when grown in pairs or combined, they were able to suppress this conversion (Soory and Ahmad, 1997).

One study assessed the response of *Bacillus cereus* strain Socransky 67 to the presence of progesterone and testosterone (Ojanotko-Harri et al., 1990). This bacterium expressed 17β-hydroxysteroid dehydrogenase activity, and thus appeared capable of metabolizing progesterone and testosterone.

Yokoyama et al. (2005) investigated the response of various periodontal pathogens when treated with estradiol or progesterone. In this study, the growth of *Campylobacter rectus* – a periodontal pathogen that has been associated with preterm low birth weight (Varadan and Ramamurthy, 2015) – was shown to be influenced by estrogen and progesterone. The presence of these SSH increased its growth similarly to *P. intermedia* as described by Kornman and Loesche (1982).

*Treponema denticola* has also received attention due to its presence in subgingival biofilms. This microorganism has the ability to 5α-reduce SSH *in vitro* (Clark and Soory, 2005). This finding is relevant specially for testosterone, whose 5α-reduced form – DHT – is a powerful androgen in the human body (Swerdloff et al., 2017). The growth of this oral spirochete can be inhibited by progesterone at concentrations found in plasma (Clark and Soory, 2006; Clark and Soory, 2007), showing that *Treponema denticola* is sensitive to changes in concentrations of SSH present in the medium.

#### 2.1.2 Caries Associated Bacteria

Ojanotko-Harri et al. investigated the metabolism of progesterone and testosterone by *Streptococcus mutans* strain Ingbritt cultures (Ojanotko-Harri et al., 1990). They showed that *S. mutans* is capable of metabolizing testosterone and progesterone by measuring the presence of metabolites in the culture medium. Based on the previously obtained results, the same group published their work on metabolism of 17β-estradiol by oral *S. mutans*, amongst other microorganisms (Ojanotko-Harri et al., 1991), showing 17β-hydroxysteroid dehydrogenase activity of this bacterium. Together, these publications are the only available evidence that *S. mutans* is capable of metabolizing SSH *in vitro*.

#### 2.1.3 Oral Health Associated Bacteria

The same study that tested the ability of *S. mutans* to metabolize 17β-estradiol also analyzed the response of *S. sanguis* (Ojanotko-Harri et al., 1991), which also showed 17β-hydroxysteroid dehydrogenase activity. This is the only available evidence supporting the hypothesis that oral-health-related bacteria are also capable of metabolizing SSH *in vitro*.

#### 2.1.4 Oral Fungi

Earlier studies have reported that *Candida* species possess estrogen (*C. albicans* and *C. glabrata*) and progesterone (*C. albicans*) binding sites (Powell et al., 1984; Skowronski and Feldman, 1989; Gujjar et al., 1997). Estrogen has been described to stimulate growth in *C. albicans*, as well as the enhancement of several virulence factors (Zhang et al., 2000). This includes the formation of hyphae (Cheng et al., 2006; Prasad et al., 2012) and expression of CDR1 and CDR2 (Krishnamurthy et al., 1998; Cheng et al., 2006; Larsen et al., 2006), important genes in drug resistance. Progesterone is known to alter gene expression, both favoring resistance to drugs (Larsen et al., 2006) as well as impairing the yeast’s ability to form biofilms, colonize and invade the host (Alves et al., 2014).

Unfortunately, most of the available evidence on *Candida* has focused on the development of vaginitis (Kinsman et al., 1988) and few reports have explored the role of SSH in the oral cavity and *C. albicans* behavior (Theaker et al., 1993; Kravtsov et al., 2014). This comes to no surprise, since fungi have been systematically excluded from oral environmental studies and little is known about their commensal behavior in contrast to their role in oral pathology (Krom et al., 2014). Until now, evidence indicates that *Candida* can benefit from the presence of SSH though in a different way than some oral bacteria do.

### 2.2 The Effect of SSH on the Host: Role in Immune Fitness

Up until now, only three studies have investigated the effect of SSH on the response of human gingival fibroblasts to oral bacteria *in vitro* (Soory, 1995; Soory and Ahmad, 1997; Yokoyama et al., 2005). The dynamics between SSH, oral bacteria and other cell types is not known.

In 1995, Soory cultured gingival fibroblasts from healthy gingival tissue in the presence of testosterone and supernatants obtained from bacterial cultures of *A. actinomycetemcomitans, P. intermedia and P. gingivalis* (Soory, 1995). The presence of these bacterial supernatants increased the cell’s ability to convert testosterone into dihydrotestosterone (DHT).

In a later study, gingival fibroblasts from chronically inflamed gingival tissue were cultured in the presence of 4-androstenedione and supernatants from the same bacterial cultures, separately (Soory and Ahmad, 1997). This interaction resulted in an increased conversion rate of 4-androstenedione to testosterone by gingival fibroblasts when grown in the presence of each bacterial culture’s supernatant.

Yokoyama et al. assessed the reaction of gingival fibroblasts in the presence of *C. rectus* in combination with estradiol (Yokoyama et al., 2005). An additive effect was observed in the production of vascular endothelial growth factor (VEGF) by these gingival fibroblasts. VEGF is a potent blood vessel function regulator that affects the endothelium, including the promotion of hyperpermeability, angiogenesis and endothelial cell growth (Connolly, 1991).

The existence of SSH metabolic pathways can have an evolutionary explanation. Host and microbiome have co-evolved and remain to do so, adapting their behavior and response to the existing circumstances. Available evidence has mostly reported effects on the growth of oral microorganisms, catabolic effects on SSH and effects on the virulence factors of the tested bacteria. This steroid use can be convenient for the microorganisms in different ways, which have been earlier described for gut bacteria, including: (1) synthesis of surface capsular proteins; (2) persistence and dissemination in their human host; (3) co- and inter-aggregation and; (4) production of energy *via* electron transfer chain (Bokkenheuser et al., 1983). It has also been proposed that steroid metabolites that result from the reduction of SSH by some bacteria could serve as nutritional requirements for themselves and other microorganisms as well as enabling synthesis of matrices associated with host evasion mechanisms (Soory, 2000).

Since the beginning of the exploration of the effect of SSH on oral bacteria and fungi, it has repeatedly been observed that a number of sub- and supragingival bacteria are capable of changing their behavior in the presence of these molecules. This has also been observed for the fungus *Candida*. The different responses triggered by SSH not only in disease-associated bacteria but also health-associated and commensal microorganisms hint a versatile influence of SSH in an oral microcosm.

Changes in the metabolism of fibroblasts have also been documented. This has shown that SSH are not only capable of affecting the microorganisms in the environment, but may also as a result, change the response of the host.

*In vitro* studies have set the fundamentals to question the role of the oral microbiome during periods of changing hormone levels, giving enough reason to presume that not only inflammation and changes in the immune response of the host are responsible for the observable clinical changes. As explained above, there are unfortunately no *in vitro* studies that have tested the response of an oral microcosm to the presence of SSH. The hypothesis of changes in a polymicrobial setting have only been investigated *in vivo* and these results will be discussed below.

## 3 *In Vivo* Studies

Since gingival alterations during pregnancy were linked to hormonal changes (Ziskin, 1938; Ziskin and Nesse, 1946) different factors have been pinpointed as contributors to this phenomenon. Immunological, vascular, cellular and microbiological changes have been and continue to be studied and until today, results involving the oral microbiome remain contradictory.

The contribution of female SSH to the alteration of periodontal bacteria has been thoroughly reviewed (Kumar, 2013) and it was concluded that evidence remains insufficient. Even though studies suggest a preferential colonization by specific periodontal bacteria during periods of fluctuating hormones, several confounding factors make it difficult to positively link these changes to a selective enhancement of the subgingival microbiome.

This, paired with differences in study design and microbiological analysis methods have made interpretation of the results a challenging task. However, since the publication of the aforementioned review, several new studies have been published. Recent studies have focused on endogenous fluctuations (physiological and pathological) as well as exogenous administration of hormones. Species affected during these changes are listed on **Tables 2**–**4**, and the corresponding studies are discussed below.

**Table 2:** Bacteria and fungi affected by the menstrual cycle, pregnancy and postpartum [Not included in (Kumar, 2013)].

Species	Reference	Study design	Sampling	Control	Hormone measurements	Clinical assessment	Identification method	Results
***Actinobaculum* spp.**	(Balan et al., 2018)	CS	US	No	No	GBI, PI	16S rRNA sequencing	Significant reduction in the post-partum period compared with the three trimesters of pregnancy
			SGP					
***Aggregatibacter actinomycetem-comitans***	(Borgo et al., 2014)	L	SGP	Yes	No	BOP, CAL, GBI, PD, VPI,	qPCR	Higher gingival inflammation and higher presence on 2^nd^ and 3^rd^ trimester
	(Fujiwara et al., 2017)	L	US	Yes	No	No	PCR	Higher incidence during 1^st^ and 2^nd^ trimester
			SGP					
	(Balan et al., 2018)	CS	US, SGP	No	No	GBI, PI	16S rRNA sequencing	Significantly associated with pregnancy gingivitis
***Actinomyces* spp.**	(Paropkari et al., 2016)	CS	SGP	Yes	No	No	16S-pyrotag sequencing	Significantly decreased in the pregnant group
***Campylobacter***	(Bostanci et al., 2021)	L	SS	No	Yes^∇^	No	NGS	Abundance varied through the menstrual cycle
***Campylobacter rectus***	(Balan et al., 2018)	CS	US, SGP	No	No	GBI, PI	16S rRNA sequencing	Significantly associated with pregnancy gingivitis
***Enterobacter* spp.**	(Paropkari et al., 2016)	CS	SGP	Yes	No	No	16S-pyrotag sequencing	Highly present in estrogen rich environments. Correlated to estrogen-metabolising spp.
***Fusobacterium nucleatum***	(Balan et al., 2018)	CS	US, SGP	No	No	GBI, PI	16S rRNA sequencing	Significantly associated with pregnancy gingivitis
***Haemophilus***	(Bostanci et al., 2021)	L	SS	No	Yes^∇^	No	NGS	Abundance varied through the menstrual cycle
***Methylo-Bacterium* spp.**	(Paropkari et al., 2016)	CS	SGP	Yes	No	No	16S-pyrotag sequencing	Highly present in estrogen rich environments. Correlated to estrogen-metabolising spp.
***Neisseria* spp.**	(Paropkari et al., 2016)	CS	SGP	Yes	No	No	16S-pyrotag sequencing	Significantly decreased in the pregnant group. Related to gingival inflammation
	(Lin et al., 2018)	L	US	Yes	Yes**	GI, PLI, SBI	16S rRNA sequencing	Significantly increased in the pregnant group
			SupGP					
***Oribacterium***	(Bostanci et al., 2021)	L	SS	No	Yes^∇^	No	NGS	Abundance varied through the menstrual cycle
***Prevotella intermedia***	(Emmatty et al., 2013)	CS	SGP	Yes	No	GI, PD, PI	Culture	Higher GI and higher *P.i.* counts on 2^nd^ and 3^rd^ trimester
	(Balan et al., 2018)	CS	US, SGP	No	No	GBI, PI	16S rRNA sequencing	Significantly associated with pregnancy gingivitis
***Prevotella nigrescens***	(Machado et al., 2016)	L	SupGP	No	No	BOP, CAL, PC, PD	FISH	Decrease over the course of pregnancy and post-partum
			SGP					
***Prevotella* spp.**	(Balan et al., 2018)	CS	US	No	No	GBI, PI	16S rRNA sequencing	Significant reduction in the post-partum period compared with the three trimesters of pregnancy
			SGP					
	(Bostanci et al., 2021)	L	SS	No	Yes^∇^	No	NGS	Abundance varied through the menstrual cycle
	(Lin et al., 2018)	L	US	Yes	Yes**	GI, PLI, SBI	16S rRNA sequencing	Positive correlation with hormonal changes
			SupGP					
	(Paropkari et al., 2016)	CS	SGP	Yes	No	No	16S-pyrotag sequencing	Highly present in estrogen rich environments. Correlated to estrogen-metabolising spp.
***Pseudomonas* spp.**	(Paropkari et al., 2016)	CS	SGP	Yes	No	No	16S-pyrotag sequencing	Highly present in estrogen rich environments. Correlated to estrogen-metabolising spp.
***Porphyromonas* spp.**	(Lin et al., 2018)	L	US	Yes	Yes**	GI, PLI, SBI	16S rRNA sequencing	Significantly increased in the pregnant group
			SupGP					
***Porphyromonas gingivalis***	(Fujiwara et al., 2017)	L	US	Yes	No	No	PCR	Higher incidence during 1st and 2nd trimester
			SGP					
	(Massoni et al., 2019)	CS	SGP	Yes	Yes*	CAL, GI, PD, PI	qPCR	Positively correlated with progesterone levels
	(Balan et al., 2018)	CS	US, SGP	No	No	GBI, PI	16S rRNA sequencing	Significantly associated with pregnancy gingivitis
***Streptococcus* spp.**	(Paropkari et al., 2016)	CS	SGP	Yes	No	No	16S-pyrotag sequencing	Highly present in estrogen rich environments. Correlated to estrogen-metabolising spp.
***Tannerella forsythia***	(Massoni et al., 2019)	CS	SGP	Yes	Yes*	CAL, GI, PD, PI	qPCR	More frequently observed during 1^st^ trimester. Associated to gingivitis cases in pregnant women.
***Treponema* spp.**	(Lin et al., 2018)	L	US	Yes	Yes**	GI, PLI, SBI	16S rRNA sequencing	Significantly increased in the pregnant group. Positive correlation with hormonal changes
			SupGP					
***Vagococcus* spp.**	(Paropkari et al., 2016)	CS	SGP	Yes	No	No	16S-pyrotag sequencing	Highly present in estrogen rich environments. Correlated to estrogen-metabolising spp.
***Veillonella* spp.**	(Paropkari et al., 2016)	CS	SGP	Yes	No	No	16S-pyrotag sequencing	Significantly decreased in the pregnant group
***Veillonella parvula***	(Balan et al., 2018)	CS	US	No	No	GBI, PI	16S rRNA sequencing	Significant reduction in the post-partum period compared with whole pregnancy
			SGP					
***Candida* spp.**	(Fujiwara et al., 2017)	L	US	Yes	No	No	PCR	Higher incidence during 2^nd^ and 3^rd^ trimester
			SGP					

BOP, bleeding on probing; CAL, clinical attachment level; CS, cross-sectional; GBI, gingival bleeding index; GI, gingival index; L, longitudinal; NGS, next generation sequencing; OPG, orthopantomography; PC, presence of calculus; PD, probing depth; PI, plaque index; PLI, plaque index; (q)PCR, (quantitative) polymerase chain reaction; SBI, sulcus bleeding index; SGP, sub gingival plaque; SupGP, supra gingival plaque; SS, stimulated saliva; US, unstimulated saliva; VPI, visible plaque index; ^∇^plasma estradiol and progesterone; *serum estradiol and progesterone; **salivary estradiol and progesterone.

**Table 3:** Bacteria and fungi affected by polycystic ovary syndrome (PCOS).

Species	Reference	Study design	Sampling	Control	Hormone measurements	Clinical assessment	Identification method	Results
***Actinobacteria***	(Lindheim et al., 2016)	CS	US	Yes	Yes*	No	16S rRNA sequencing	Decreased relative abundance
***Fusobacterium nucleatum***	(Akcalı et al., 2014)	CS	BS, US	Yes	No	BOP, PD, PI	qPCR	Bacterial counts significantly higher in women with PCOS and gingivitis.
							ELISA	
***Peptostrepto-coccus micros***	(Wendland et al., 2020)	CS	SGP	Yes	Yes**	BOP, GI, PD, PLI	PCR	Decreased in subjects with PCOS
***Prevotella intermedia***	(Akcalı et al., 2014)	CS	BS, US	Yes	No	BOP, PD, PI	qPCR	Bacterial counts significantly higher in women with PCOS and gingivitis.
							ELISA	Increased serum antibody levels to P. intermedia in the presence of PCOS
***Porphyromonas gingivalis***	(Akcalı et al., 2014)	CS	BS, US	Yes	No	BOP, PD, PI	qPCR	Bacterial counts significantly higher in women with PCOS and gingivitis.
							ELISA	Increased serum antibody levels to P. gingivalis in the presence of PCOS
***Streptococcus oralis***	(Akcalı et al., 2014)	CS	BS, US	Yes	No	BOP, PD, PI	qPCR	Bacterial counts significantly higher in women with PCOS and gingivitis.
							ELISA	Increased serum antibody levels to S. oralis in the presence of PCOS
***Tannerella forsythia***	(Akcalı et al., 2014)	CS	BS, US	Yes	No	BOP, PD, PI	qPCR	Bacterial counts significantly higher in women with PCOS and gingivitis
							ELISA	
***Treponema denticola***	(Wendland et al., 2020)	CS	SGP	Yes	Yes**	BOP, GI, PD, PLI	PCR	Decreased in subjects with PCOS

BOP, bleeding on probing; BS, blood serum; PD, probing depth; PI, plaque index; PLI, plaque index; (q)PCR, (quantitative) polymerase chain reaction; SGP, sub gingival plaque; *Serum estrone, 17-estradiol, total testosterone, androstenedione, DHEA, DHEA-S and DHT; **Serum insulin, FSH, LH, total testosterone, 17-β-estradiol, DHEA-S, and SHBG.

**Table 4:** Bacteria and fungi affected by exogenous administration of SSH [Not included in (Kumar, 2013)].

	HTR								Androgen abuse							
Species	Reference	Study design	Sampling	Control	Hormone measurements	Clinical assessment	Identification method	Results	Reference	Study design	Sampling	Control	Hormone measurements	Clinical assessment	Identification method	Results
***Aggregatibacter actinomycetem-comitans***	Unknown								(Brusca et al., 2014)	CS	SGP	Yes	No	GI, PI, PD, CAL, number of teeth, radiographs	Culture	AAS users presented significantly higher proportions of *A.a., P.i.* and *P.g.* compared to controls and higher GI and CAL.
***Prevotella intermedia***	(Tarkkila et al., 2010)	L	SGP	Yes	No	CPITN OPG	qPCR	Lower incidence in HRT users after 2 years								
***Porphyromonas gingivalis***	Unknown															
***Tannerella forsythia***	(Tarkkila et al., 2010)	L	SGP	Yes	No	CPITN OPG	qPCR	Lower incidence in HRT users after 2 years	Unknown							
***Candida albicans***	Unknown								(Brusca et al., 2014)	CS	SGP	Yes	No	GI, PI, PD, CAL, number of teeth, radiographs	Culture	Negatively affected by AAS
***Candida dubliniensis***																Negatively affected by AAS
***Candida* species**																AAS users: higher proportions of *C. parapsilosis* and *C. tropicalisis.*
																Higher GI and CAL

CS, cross-sectional; L, longitudinal; SGP, sub gingival plaque; CAL, clinical attachment level; CPTIN, community periodontal index of treatment needs; GI, gingival index; PI, plaque index; PD, probing depth; OPG, orthopantomography; qPCR, quantitative polymerase chain reaction.

### 3.1 Endogenous Fluctuations of Sex Steroid Hormones and the Oral Microbiome

Physiological and physical changes taking place during lifetime are regulated by various biological processes, including important fluctuations in SSH. These fluctuations occur differently depending on the sex of the individual. While females experience marked increases or decreases of SSH -especially during pregnancy- males face progressive and steady changes (Jones and Boelaert, 2015). This could explain why studies assessing the effects of SSH on the oral cavity and oral microorganisms have mainly focused on females of reproductive age. A limited number of studies have included males as study subjects (Kumar, 2013) representing an important shortcoming. Recent studies have addressed the effect of SSH fluctuations on the oral microbiome during the menstrual cycle, pregnancy and postpartum, and these are presented below. The existing studies focusing on changes during puberty have already been reviewed by Kumar and thus have not been included in this review.

#### 3.1.1 Menstrual Cycle

The menstrual cycle is a periodic shift in hormone production paired with physical changes of the uterus and ovaries in women of reproductive age (Reed and Carr, 2000; Bates and Bowling, 2013). These cyclic changes take place in 25–30-day periods and can be divided into two phases: (1) a follicular or proliferative phase, and (2) a luteal or secretory phase (Reed and Carr, 2000). The follicular phase starts with the first day of the menstrual period and ends with the ovulation, understood as the release of a mature egg from the ovary. The luteal phase, starts with the ovulation and ends just before the menstrual period (Bates and Bowling, 2013). During the follicular phase, estrogen levels in blood steadily increase until just before ovulation, reaching between 130 and 200 pg/mL from an initial concentration of approximately 50 pg/mL (Saucier, 2009). After ovulation, the luteal phase begins and progesterone levels in blood start to increase (Bates and Bowling, 2013), raising from less than 1 ng/mL to values between 10-35 ng/mL (Taraborrelli, 2015). If the egg is not fertilized, both estrogen and progesterone will sharply decrease to baseline levels, leading to the menstrual period (Bates and Bowling, 2013).

A limited number of studies have investigated the effects of the menstrual cycle on both gingival and oral microbiological changes, without reaching definite consensus (Kumar, 2013). This could be explained by small sample-size studies and methodologies that either targeted specific oral species or provided non-specific information about microbiological changes during the menstrual cycle (Prout and Hopps, 1970; Jensen et al., 1981; Calil et al., 2008; Fischer et al., 2008). To overcome this the use of new techniques can provide us with a better understanding of the influence of the menstrual cycle on the oral microbiome. A recently published study, investigated this using next generation sequencing (Bostanci et al., 2021). This study reported stability of the oral microbiome through the menstrual cycle of 103 women of reproductive age. However, they did observe variations in abundance of certain genera during the menstrual cycle (e.g., *Campylobacter, Haemophilus, Prevotella* and *Oribacterium*) and a higher diversity of species during the luteal phase. These results support the hypothesis that periodic hormonal shifts can influence the oral microbiome, but more studies are needed to confirm this and assess the possible clinical implications.

#### 3.1.2 Pregnancy and Post-Partum

During the course of pregnancy, the female body experiences several physiological and physical changes. Estradiol and progesterone play a major role during pregnancy. Both are produced by the corpus luteum until the 6^th^ – 7^th^ week of pregnancy after which the production is taken over by the placenta. Estrogen increases steadily until delivery, rising from 0.1 ng/mL during the follicular phase of a normal menstrual cycle to 6-30 ng/mL at the end of the third trimester of pregnancy. Progesterone rises from less than 1 ng/mL during the follicular phase to 100-300 ng/mL at term (Tal et al., 2000). These values decrease back to normal levels at post-partum (Raber-Durlacher et al., 1994).

These considerable fluctuations have been linked to clinical changes in the periodontium: estradiol has been reported to increase gingival inflammation and progesterone to increase vascular permeability (Güncü et al., 2005). However, these direct effects on the oral tissues may not be the only ones. Based on the available *in vitro* evidence, it becomes apparent that hormones can directly and indirectly affect the oral microbiome.

Recent studies have mostly targeted specific species known for their affinity for SSH by means of selective media, PCR, qPCR and Fluorescence *in situ* hybridization (FISH). Two studies have analyzed the microbiome in depth by means of sequencing techniques.

*In vitro* studies have not reported any effect of either estrogen or progesterone on *Aggregatibacter actinomycetemcomitans*. However, this bacterium appears to be more prevalent during the second and third trimester of pregnancy according to Borgo et al. (2014) and during the first and second trimester according to Fujiwara et al. (2017). Even though both studies had a longitudinal design and used PCR techniques, the sample sizes varied greatly (9 and 125 subjects, respectively).

A cross-sectional study reported increased counts of *Prevotella intermedia* paired with increased gingival inflammation during the 2^nd^ and 3^rd^ trimester of pregnancy (Emmatty et al., 2013), confirming similar results earlier documented (Kornman and Loesche, 1980; Raber-Durlacher et al., 1994). *In vitro* studies also support these observations, given that estrogen and progesterone promote *P. intermedia’s* growth (Kornman and Loesche, 1982; Fteita et al., 2014). Nevertheless, this was not observed on a recent longitudinal study, where control subjects showed higher *P. intermedia* counts instead (Borgo et al., 2014).

During a longitudinal study, *Prevotella nigrescens* was noted to decrease significantly from pregnancy to post-partum (Machado et al., 2016). A high variability between pregnancy and post-partum was reported for other bacteria (*Fusobacterium nucleatum* and *Prevotella intermedia*) but not to a significant level. This could be related to the small sample size and a high drop-out rate. *In vitro, P. nigrescens* shows enhanced biofilm formation at estradiol concentrations present during the second trimester of pregnancy and enhanced planktonic growth at estradiol concentrations corresponding to the third trimester of pregnancy (Fteita et al., 2014). Based on this, it seems logical that after estradiol levels have dropped, *P. nigrescens’* counts decline.

Increased microbial counts of *Porphyromonas gingivalis* have been reported during the first and second trimester of pregnancy compared to non-pregnant controls (Fujiwara et al., 2017). Also, a correlation between bacterial counts and progesterone levels in the first trimester of pregnancy has been observed (Massoni et al., 2019). *In vitro* studies have reported effects of estrogen and progesterone on *P. gingivalis*, which could partially explain this observation (Kornman and Loesche, 1982).

A higher prevalence of *Tannerella forsythia* has been noted to take place during the first trimester of pregnancy. This bacterium, during the same study, also correlated with the diagnosis of gingivitis among all pregnant participants independent of the pregnancy stage (Massoni et al., 2019). Unfortunately, no *in vitro* studies have reported effects of SSH on *T. forsythia.*

Fujiwara et al., also reported a significant increase of *Candida* species during the second and third trimester (Fujiwara et al., 2017). These results suggest an increased susceptibility during pregnancy for the colonization of certain periodontal bacteria and *Candida* species, which has also been observed *in vitro* (Powell et al., 1984; Skowronski and Feldman, 1989; Gujjar et al., 1997).

Studies using a more comprehensive approach to assess the effects on the oral microbiome have been recently carried out.

So far, three studies using sequencing techniques have been published and results show that microbial shifts occur during periods of SSH fluctuations and not only periodontal species are involved, but also novel phylotypes.

One cross-sectional study assessed the subgingival flora of 44 pregnant and non-pregnant women (smokers and non-smokers) using 16S-pyrotag sequencing, to investigate the synergistic effects of smoking during pregnancy (Paropkari et al., 2016). A significant clustering of pregnancy and smoking status was observed, indicating a different effect on the subgingival microbiome for each condition alone and combined. Results also indicated that pregnancy promoted the growth of Gram-negative facultative anaerobes to the detriment of anaerobes. This does not agree with previous studies (Gürsoy et al., 2009; Emmatty et al., 2013) which could be partially explained by the inclusion of only periodontally healthy participants. Additionally, a co-occurrence network analysis –aimed to visualize possible relations between bacteria based on environmental factors – showed that among others, *Pseudomonas, Prevotella, Methylobacterium, Vagococcus* and *Enterobacter* are highly present in estrogen rich environments and strongly correlated to species known to metabolize estrogen. Therefore, despite the small sample size, this study provides insight into unexplored species that might also be affected by the presence of SSH.

A second cross-sectional study aimed to present a snapshot of the oral microbiome during all trimesters of pregnancy and post-partum using 16S rRNA gene sequencing (Balan et al., 2018). Diversity remained stable across pregnancy and post-partum whereas an increase in pathogenic species during pregnancy was documented as well as a marked decrease after delivery with a reestablishment of oral health. An interesting observation was the co-occurrence of gingival bleeding and novel phylotypes rather than the usually-associated species.

Only one longitudinal study has used sequencing methods to assess the oral microbiome of pregnant women (Lin et al., 2018). Unlike most studies, supragingival samples were used for the microbiological analysis and hormonal levels were also assessed.

It was reported that several genera are responsible for the clustering according to the pregnancy trimester and that different genera were positively correlated with the surge in SSH, including *Prevotella, Treponema, Cardiobacterium, Aggregatibacter, Leptotrichia* among others. Even though the study sample was small, a shift could be observed, suggesting that hormonal changes during pregnancy could push the biofilm towards an unbalanced or dysbiotic state.

#### 3.1.3 Menopause

Two cross-sectional studies have explored the microbial composition of post-menopausal women (Brennan et al., 2007; Hernández-Vigueras et al., 2016). Even though both studies reported the co-occurrence of a similar group of periodontal pathogens, lack of follow-up and different inclusion criteria together with the absence of hormonal measurements makes drawing conclusions difficult.

### 3.2 Anomalous Fluctuations of SSH

Polycystic ovary syndrome (PCOS) is an endocrine, reproductive and metabolic condition affecting women of reproductive age, with a prevalence between 15-20% (Sirmans and Pate, 2013). Recently, it was reported that individuals suffering from PCOS may also experience a higher prevalence and risk of developing periodontal disease (Machado et al., 2020). Subsequently, microbiological changes have been pointed as one of the causes (Akcalı et al., 2014; Lindheim et al., 2016; Wendland et al., 2020). To date, three studies have investigated changes in oral bacterial species in individuals suffering from PCOS. In the first study, salivary samples from individuals with PCOS (with and without gingivitis) were compared to a matched control group, using PCR for bacterial identification (Akcalı et al., 2014). It was observed that *F. nucleatum, P. gingivalis, P. intermedia*, *S. oralis* and *T. forsythia* were significantly higher in individuals presenting PCOS and gingivitis, compared to controls. Also, serum antibody levels were measured using ELISA. Individuals with PCOS and gingivitis showed an increased antibody-mediated immunological response to *P. gingivalis*, and periodontally healthy individuals with PCOS, showed an increased response to *P. intermedia* and *S. oralis*.

One more study used PCR to evaluate the presence of nine different periodontal bacteria in young individuals with and without PCOS (Wendland et al., 2020). In this study, no particular microbiome alterations could be linked to PCOS. Interestingly, the control group showed a significantly higher prevalence of pathogenic and potentially pathogenic bacteria. These results could have been affected by a small sample size and less strict diagnostic criteria for the selected population. Nevertheless, this is the only study done in young individuals (2 years after the onset of menstruation) and more studies are needed to confirm these observations.

To date, only one study has used next generation sequencing to investigate possible changes in the oral microbiome of individuals with PCOS (Lindheim et al., 2016). This study reported a decreased relative abundance of the phylum *Actinobacteria* without further significant variation in community composition and diversity between the studied groups. This reduction could favor an environment suitable for disease-associated bacteria, increasing the risk of dysbiosis. A small sample size and the absence of more severe forms of PCOS could have influenced the obtained results.

Existing research remains contradictory regarding the role of PCOS in the composition of the oral microbiome and its contribution to inflammation and periodontal health/disease.

### 3.3 Exogenous Administration of Sex Steroid Hormones

#### 3.3.1 Hormonal Contraception

Hormonal contraceptive methods consist of synthetic forms of progestagens and/or estrogens at levels aimed to suppress ovulation, similar to the ones achieved during pregnancy. These come in different dosages and formulations and can be administered orally (oral contraceptive pills), in the form of injections, implants, intravaginal, topical patches and intra uterine devices (Colquitt and Martin, 2015).

Previous studies have investigated the possible influence of oral contraceptives (OC) on the composition of the subgingival microbiome (Jensen et al., 1981; Klinger et al., 1998). These studies have been reviewed by Kumar (2013) and even though results indicate that OC promote a selection of certain periodontal microorganisms, the applicability of these conclusions today is not feasible. Differences in the formulation, dosage and routes of administration of contraceptives as well as changes these have undergone through the years, make standardization nor generalization possible.

Higher counts of *P. gingivalis, P. intermedia* and *A. actinomycetemcomitans* have been observed in women using oral contraceptives together with an increased risk of developing periodontitis (Brusca et al., 2010). *Candida* species have also been reported to increase in counts for individuals using OC for at least three years (Brusca et al., 2010). In this recent publication smokers were included as participants but not separately analyzed. It is well known that tobacco negatively affects oral health and the oral microbiome resulting in an increased risk of developing periodontal disease (Gloria et al., 2002; Mason et al., 2015) and this must be considered when weighing these results.

The existing evidence points towards a role of oral contraceptives on the composition of the subgingival microbiome. Nevertheless, more studies assessing this interaction and taking into account the different formulations, dosages and administration routes are needed.

#### 3.3.2 Hormone Replacement Therapy

HRT is the treatment of choice for acute climacteric syndrome and to prevent long-term estrogen deficiency. It consists of single (estrogen) or combined (estrogen-progesterone) preparations. Administration can be oral, transdermal, percutaneous, intramuscular, intranasal or local with dosing and timing depending on the case (Fait, 2019).

Only one study has investigated the effect of HRT on oral health. Samples were taken at baseline and after two years of therapy (Tarkkila et al., 2010). Both clinical and microbiological parameters were evaluated and it was concluded that the use of HRT does not have a significant effect on the overall composition of the oral microbiome. Additionally, there was a decrease of certain species usually found in periodontal disease – namely *P. gingivalis, P. intermedia* and *T. forsythia* – after 2 years of HRT use. Even though these results seem optimistic and could indicate a positive influence of HRT on periodontal health, the role of HRT on clinical attachment loss (CAL) remains debatable and inconclusive (Chaves et al., 2020).

More studies are needed to assess the risks and benefits of undergoing HRT from an oral health perspective.

#### 3.3.3 Androgen Use

Androgens – also known as male steroid hormones – include testosterone and its metabolites as well as synthetic preparations. Synthetic preparations are largely used to treat androgen deficiency but these are also (mis)used by individuals looking forward to gaining muscular mass, leading to different complications (Sadaie et al., 2018).

Studies evaluating oral health and the use of androgens are scarce. The reason behind this could be that androgens do not experiment extreme fluctuations like female SSH do. This results in a more stable concentration throughout the life of an individual (Harman et al., 2001).

It can be expected that androgens have an effect in the oral cavity as androgen receptors have been described in diverse oral tissues such as gingiva (Southren et al., 1978), oral mucosa (Ojanotko-Harri et al., 1992) and salivary glands (Laine et al., 1993) indicating that they can also act as modulating agents in the oral cavity. Earlier reports have noted that DHT can be metabolized by oral fibroblasts resulting in a collagen matrix stimulatory effect (Soory and Ahmad, 1997). This is consistent with later studies reporting that individuals consuming anabolic steroids experience gingival inflammation compared to controls (Ozcelik et al., 2006). One study investigated the effect of anabolic steroids on the oral microbiota of a group of body builders (Brusca et al., 2014). Results showed that users had a significantly higher prevalence of severe periodontal disease, higher gingival inflammation and attachment loss as well as a higher prevalence of *A. actinomycetemcomitans, P. intermedia, P. gingivalis* and *Candida* species associated to a diseased oral state, compared to a control group of anabolic steroids non-users.

Taken together, the above-described studies indicate that it is likely that SSH affect the oral cavity, however more studies are needed to understand how endogenous and exogenous androgens can affect the balance between the host and the oral microbiome.

## 4 Discussion

We set out to review the data available on the effect of SSH in the oral cavity. Various *in vitro* and *in vivo* studies have aimed to clarify the direct and indirect effects of SSH on the oral microbiota and host tissues. It is important to note that both these approaches have advantages and limitations. *In vitro* studies provide a controlled environment and can explain the mechanisms involved in microbial responses to SSH. Nonetheless, they provide only a simplified interpretation of reality and lack important factors that play a role in the complex host-microbiome interaction. And while *in vivo* studies offer real-life conditions whose results can be directly applicable to the clinical field, there is a high degree of subject variability and, more importantly, a real risk of bias. Both *in vitro* and *in vivo* studies have mostly targeted specific periodontal pathogens and failed to look at the oral microbiome in its totality. The incorporation of both approaches is therefore most suitable to grasp the whole picture.

The great variability between the methodologies applied to date in *in vivo* and *in vitro* studies makes them difficult to compare. The technologies used to classify microorganisms in early studies were less sensitive and did not document all species correctly.

The available evidence makes it evident that hormones can affect the oral microbiome both directly and indirectly. The remaining question is if and to what extent these effects play a role in oral health and disease and what the underlying mechanisms are.

Recent studies have approached this question using clinical evaluation paired with current technologies such as sequencing, that focus not only on classic periodontal pathogens but also on the oral microbiome (Balan et al., 2018; Lin et al., 2018; Bostanci et al., 2021). These analyses have yielded interesting results regarding the diversity of the oral microbiome and how SSH can promote a shift in its composition that is not exclusive to known periodontal pathogens (Short et al., 2014).

Building on the available evidence, we propose a hypothesis that describes the mechanism of this interplay between SSH, host and microbiome.

By metabolizing SSH, certain bacteria can directly and indirectly influence SSH concentration in the environment (Kornman and Loesche, 1982; Soory and Ahmad, 1997). Their conversion into more active molecules (i.e. by forming DHT from testosterone by means of 5α-reduction) can directly affect oral tissues. Bacterial enzymes can also stimulate the natural conversion of testosterone into DHT by oral fibroblasts (Soory, 1995). While this bidirectional interaction has been proposed only for certain bacterial species, we believe that it is not an exclusive response of a limited number of microorganisms, and more generally occurs in the oral microbiome. Specifically, this interaction evidences a bidirectional response, supporting the hypothesis that biofilm-mediated hormonal metabolism can result in a cellular response.

**Figure 2:**
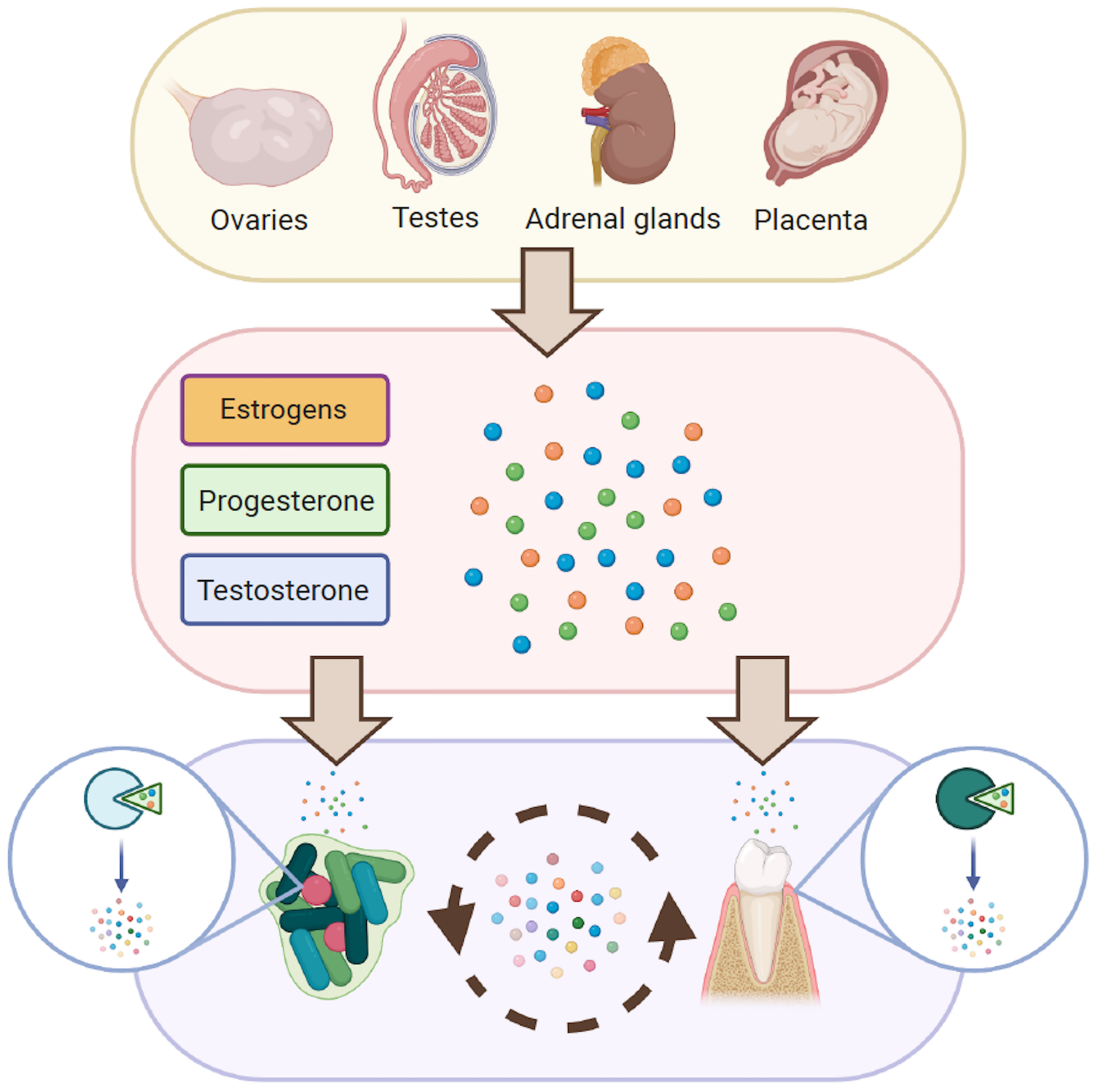
Proposed interaction between SSH, the host and its microbiome. Solid arrows represent known interactions between SSH, oral microorganisms and oral tissues. Dashed arrows represent hypothesized interactions.

We therefore propose a hypothesis to explain how SSH concentrations in saliva can lead to shifts in the oral microbiome and the oral milieu that directly induce a response from the host. These shifts include the proliferation of certain species and the metabolism of SSH into other molecules. Meanwhile, the host responds to these changes and – in turn – metabolizes SSH in the medium, adapting its physiological response to the environmental conditions (**Figure 2**). This interaction generates a local feedback loop whose consequences are yet to be investigated.

There is evidence of a bidirectional interaction between hormone-metabolizing as well as producing bacteria and the host in gut bacteria (Neuman et al., 2015; Tetel et al., 2018). In rats it has been documented that *Toxoplasma gondii* – a protozoan parasite – can induce the steroidogenesis of testosterone, supporting the hypothesis that microorganisms can also modulate the synthesis of SSH to their own benefit (Lim et al., 2013).

To conclude, *in vitro* and *in vivo* studies support a direct and indirect effect of SSH on oral microbes during hormonal shifts. The classical perspective has tried to link presence of specific species to a surge or decline in SSH and subsequently to clinical changes in the periodontium. Even though this has contributed to our understanding of this complex interaction, newer studies considering the dynamics between the oral microbiome and the host are needed to grasp the underlying mechanisms. Emerging research in endocrinology suggests that there is an active and bidirectional interaction between the host and its microbiome, mediated by SSH. A fuller understanding of these events will offer immense possibilities in the treatment and prevention of disease.

## 5 Final Remarks and Future Perspectives

Unlimited perspectives are yielded by the emerging understanding of the oral microbiome as an active player in some of the host’s vital physiological processes. These microbial communities have co-evolved – and continue to co-evolve – with the human host, developing mechanisms that are mutually beneficial. This interaction, when balanced, prevents a drift towards a dysbiotic or pathological state.

To date, there is sufficient ground for stating that the presence of SSH influences the oral milieu and its inhabitants. Nevertheless, it is yet to be discovered if and how this is involved in the maintenance of an oral ecological balance.

Hormone concentrations in saliva can be altered during periods of intrinsic SSH hormone fluctuations such as puberty, menstrual cycle, pregnancy, or by extrinsic fluctuations such as the use of oral contraceptives, hormone replacement therapy, gender affirming therapy or androgen use. By contributing to our understanding of oral health and how to maintain it, a better understanding of the role of SSH in the oral cavity will allow clinicians to deliver more personalized care for their patients.

## Author Contributions

PC proposed the topic to be reviewed, performed the search, extracted and analyzed the data and wrote and edited the original draft. BK and MV reviewed and edited the original draft and supervised the work. All authors contributed to the article and approved the submitted version.

## Funding

The research was funded by the Academic Centre for Dentistry Amsterdam (ACTA), University of Amsterdam and VU University Amsterdam.

## Conflict of Interest

The authors declare that the research was conducted in the absence of any commercial or financial relationships that could be construed as a potential conflict of interest.

## Publisher’s Note

All claims expressed in this article are solely those of the authors and do not necessarily represent those of their affiliated organizations, or those of the publisher, the editors and the reviewers. Any product that may be evaluated in this article, or claim that may be made by its manufacturer, is not guaranteed or endorsed by the publisher.
