# Supplementary material for: Sex Steroid Hormones as a Balancing Factor in Oral Host Microbiome Interactions
Source: Front Cell Infect Microbiol. 2021 Sep 29;11:714229. doi: 10.3389/fcimb.2021.714229 (PMC8511811; doi:10.3389/fcimb.2021.714229)
Supplement: Supplementary file 1 [file Table_1.docx]

# **Search strategy**

This review was written according to the Preferred Reporting Items for Systematic reviews and Meta-Analyses extension for Scoping Reviews (PRISMA-Scr) checklist and explanation [95]. A search was performed in the bibliographic database PubMed from inception to May 2^nd^ 2020. Search terms included controlled terms (MeSH-terms) as well as free text terms. The following terms (as well as closely related terms and synonyms) were used: ‘SSH’ and ‘oral bacteria’. No filters were used. In PubMed the following search blocks were combined: (oral OR dental OR periodontal OR periodontium OR mouth) AND (bacteria OR fungi OR yeast OR Porphyromonas OR Treponema OR candida OR Prevotella OR campylobacter OR bacteroides OR aggregatibacter OR actinomyces OR streptococcus OR bacillus OR fusobacterium) AND (sex hormones OR SSH OR SSH OR hormones OR hormonal OR pregnancy OR menopause OR puberty OR menstrua* OR hormone OR estradiol OR estradiol OR estrogen OR estrogen OR estriol OR oestriol OR testosterone OR androgen* OR progestagen* OR progesterone OR dihydrotestosterone OR androstenedione).

## **Screening process and inclusion criteria**

All search results were imported in EndNote and screened by P.C. who selected the suitable references based on their title and abstract. Full text manuscripts were downloaded via Scopus, Science Direct, Endnote, Google Scholar, Research Gate or by direct correspondence with the authors. The selected studies were once again reviewed for inclusion based on the full text. The selection criteria were as follows: (1) microorganism present in the oral cavity; (2) interaction of oral microorganisms with SSH; (3) *in vitro* studies; (4) clinical studies assessing microbial changes during puberty, menstrual cycle, pregnancy, post-partum menopause, use of contraceptive methods, use of HRT, use of androgens, use of GAHT; (5) studies published in English, Spanish, Dutch, German or French. Reference list checks were also performed, known as ‘snowballing’.
