# Supplementary material for: Sex Steroid Hormones as a Balancing Factor in Oral Host Microbiome Interactions
Source: Front Cell Infect Microbiol. 2021 Sep 29;11:714229. doi: 10.3389/fcimb.2021.714229 (PMC8511811; doi:10.3389/fcimb.2021.714229)
Supplement: Supplementary file 2 [file Table_2.docx]

# **Supplementary tables**

**Table 1.** Studies that have tested the response of different oral microbes to SSH *in vitro*

| Reference | Microbial species | Substrate | Results | Main conclusions |
| --- | --- | --- | --- | --- |
| (Kornman and Loesche, 1982) | *P. gingivalis, P. intermedia*, *P. melaninogenica* among others | Estradiol and Progesterone | *P. intermedia* and *P. melaninogenica* use estradiol and progesterone in absence of vitamin K.  Addition of fumarate to cultures of *P. gingivalis* and *P. melaninogenica* increased steroid uptake up to 500% and resulted in the detection of succinate. | Estradiol and Progesterone can promote the growth of *P. intermedia* and *P. melaninogenica* in the absence of vitamin K and seem to directly interact with the fumarate reductase system. |
| (Powell et al., 1984) | *C. albicans* strains, *C. glabrata* strains among others | Progesterone, Estradiol, Dihydrotestosterone (DHT), Testosterone, Corticosterone among others | *C. albicans* presents a corticosterone binding system and a specific binding system for 17β-estradiol. The last was also be identified in *C. glabrata.*  Competition for binding sites was observed between estrogenic steroids only.  *C. albicans* was also capable of incorporating 17β-estradiol in a specific manner. | Binding to estradiol could be a coincidental cross-reactivity of a  fungal steroid hormone binding system with human steroids, or may have physiologic or pathogenic significance. |
| (Kinsman et al., 1988) | *C. albicans* strains | Estradiol, Oestriol, Progesterone, Pregnanetriol and Testosterone among others | Oestriol, Pregnanediol and Pregnanetriol stimulate germination of *C. albicans****.***  LH increased germination in serum. | These findings may explain the predisposition of pregnant women to vaginal *Candida* infections especially in the 3^rd^ trimester of pregnancy when oestriol levels are high. |
| (Skowronski and Feldman, 1989) | *C. albicans* strains | Estradiol, Oestrone, Oestriol among others | *Candida albicans* expresses a cytosolic protein that specifically binds estrogens with high specificity.  There are no major changes in binding characteristics during the transformation from the yeast to the hyphal morphology. For other steroid hormones or fungal hormones no competitive activity was observed. | The presence of an estrogen binding protein in *C. albicans* suggests that estrogen levels in the host might alter the ability of *C. albicans* to cause an infection. |
| (Ojanotko-Harri et al., 1990) | *B.* *cereus* strain Socransky 67 and *S. mutans* strain Ingbritt | Progesterone and Testosterone | *B. cereus* possesses 5α-steroid hydrogenase, 3β- 17β- and 20α-hydroxysteroid dehydrogenases and steroid hydroxylases.  *S. mutans* possesses 5α- and 5β- steroid hydrogenases and 3α-17β- and 20α-hydroxysteroid dehydrogenases. | Both strains can metabolise Progesterone and Testosterone. The higher steroid metabolic activity of *B. cereus* could be explained by the location of *B. cereus* in the gingival sulcus. |

| Reference | Microbial species | Substrate | Results | Main conclusions |
| --- | --- | --- | --- | --- |
| (Ojanotko-Harri et al., 1991) | *S. mutans,*  *S. sanguis, B. cereus*and *C. albicans* | Estradiol | All tested strains metabolized 17β-estradiol. Oestrone was the main metabolic product. Only *B. cereus* converted 17β-estradiol into unidentified metabolites.  *S. sanguis* and *B. cereus* had the most active metabolism while *C. albicans* and *S. mutans* were also active. | Supragingival bacteria -and not only subgingival- are capable of metabolising 17β-estradiol. |
| (Soory, 1995) | *A. actinomycetemcomitans, P. intermedia and P. gingivalis +* subgingival plaque samples | Testosterone | There was a larger increase in DHT production over 4-androstenedione in subgingival plaque samples, cultures of *P. intermedia, A. actinomycetemcomi-tans* and *P. gingivalis* and gingival fibroblast cultures in the presence of *P. intermedia* and *P. gingivalis* supernatants | Bacterial metabolism and the effect of supernatants on human gingival fibroblasts influence the degree of inflammatory repair since DHT plays a role on matrix synthesis by fibroblasts in periodontal disease. |
| (Soory and Ahmad, 1997) | *A. actinomycetemcomitans, P. intermedia and P. gingivalis* | 4-androstenedione | There was an increase in DHT synthesis by human gingival tissue in the presence of culture supernatants (*P.i.* 87%; *P.g.* 50%; *A.a.* 6%) and cultured cell lines in the presence of culture supernatants (*P.i.* 40%; *P.g.* 35%; *A.a.* 40%). Combinations of the bacterial extracts showed intermediate or suppressor effects on DHT. | The simultaneous presence of the tested microorganisms *in vivo* could lead to a diminished healing response by human oral fibroblasts. |
| (Gujjar et al., 1997) | *C. albicans* | Estradiol | The size of the colonies was not uniformly stimulated by the presence of estradiol.  One estrogen-responsive strain was grown in a  chemically defined medium in the presence of either 17α and 17β isomers of estradiol being the 17β isomer the one that greatly promoted growth. | Estrogen response might be strain dependent. Estrogen binding protein in yeast might have higher specificity for the β isomer of estradiol.  Estradiol might be able to stimulate *Candida* growth through a pathway unrelated to the estrogen-binding protein. |
| (White and Larsen, 1997) | *C. albicans* | Estradiol, Testosterone and Cholesterol | Significant reduction of yeast cells germinating grown in stripped serum versus non-stripped.  Concentrations of 1mM of all the tested compounds resulted in a slight increase germination. Estradiol at a 100-fold lower concentration induced germination.  Apparent antagonism of Cholesterol and Estradiol when combined. | Estradiol promotes germination.  Effects of Estrogen may be antagonized by other steroid compounds.  Virulence of *Candida* associated with hyphal forms may be upregulated or downregulated depending on the hormonal milieu of the host. |

| Reference | Microbial species | Substrate | Results | Main conclusions |
| --- | --- | --- | --- | --- |
| (Zhang et al., 2000) | *C. albicans* strains | Estradiol | All tested strains grown in the presence of estradiol showed: Increased growth, increased resistance to incubation at 48°C, up-regulation of intracellular *hsp90* and increased expression of *CDR1 (*related to multidrug resistance). | It is likely that several virulence factors are induced by estradiol. |
| (Yokoyama et al., 2005) | *C. rectus, P. gingivalis, A. actinomycetemcomitans, F. nucleatum* and *P. intermedia* | Estradiol and Progesterone | Estradiol and Progesterone significantly stimulated the growth of *C. rectus* and *P. intermedia.* | Hormonal steroids may influence the microbial ecology of the gingival sulcus and be responsible for changes observed in subgingival bacterial flora of pregnant women. |
| (Clark and Soory, 2005) | *T. denticola* | Progesterone, Testosterone, Androstenedione, Cor-tisol, Corticosterone and Cholesterol | 5α reduced products were obtained from all tested compounds. *T. denticola* presented 3β- and 17β-hydroxy steroid dehydrogenase activity. | *T. denticola* can metabolise SSH. The *in vivo c*onsequences could include *T.*  *denticola* utilising host supplied steroids as growth factors and its metabolism acting as a virulence factor. |
| (Clark and Soory, 2006) | *T. denticola* | Progesterone, Testosterone, 4-Androstenedione and Cholesterol | All tested substrates can modulate the growth of *T. denticola*.  All tested steroids inhibited growth at different levels, being Progesterone the stronger inhibitor at concentrations found in human plasma.  Cholesterol can stimulate growth at concentrations found in human saliva. | SSH may inhibit growth by interference with metabolism of cholesterol.  The influence of combinations of cholesterol and hormonal steroids on the growth of *T. denticola* *in vitro* requires investigation. |
| (Cheng et al., 2006) | *C. albicans* strains | 17α-estradiol , 17β-estradiol, Ethynyl estradiol and  oestriol | 17β-estradiol and Ethynyl estradiol promoted Increase in *C. albicans* cells forming germ tubes and its length. Strain DSY654 showed significantly decreased number of germ tube-forming cells.  17α-estradiol and oestriol had no effect on germ tube formation.  Estrogen-treated strains showed increased expression of *CDR1* and of *CDR2*.  Exposure to exogenous estrogens did not significantly change the biomass of any culture. | Genes CDR1 and CDR2 are the most responsive to estrogen exposure.  This could explain how *C. albicans* germ tube formation and length can be affected.  More precise measurements are required to determine if these effects occur at physiological estrogen concentrations and contribute to the physiological balance between C. albicans and its host. |

| Reference | Microbial species | Substrate | Results | Main conclusions |
| --- | --- | --- | --- | --- |
| (Clark and Soory, 2007) | *T. denticola* | Progesterone, Testosterone, Androstene-3,17-dione, Corticosterone and Cholesterol | Cholesterol stimulated growth of *T. denticola.*  Certain hormonal steroids inhibited growth: progesterone> 4-androstenedione> testosterone> corticosterone. | It is likely that the ability of *T. denticola* to metabolise steroids is linked to susceptibility to growth modulation by steroids. |
| (Fteita et al., 2014) | *P. intermedia* group and  *F. nucleatum* | Estradiol | Positive correlation for *P. pallens* and *P. nigrescens* between levels of estradiol and numbers and protein levels.  Higher protein levels, formation of polysaccharides in the biofilm and enhanced coaggregation with *F. nucleatum* for both strains of *P. intermedia.* | Estradiol regulates planktonic growth, coaggregation, polysaccharide production and biofilm formation characteristics of *P. intermedia, P. nigrescens* and *P. pallens* differently. |
| (Fteita et al., 2015) | *P. intermedia* group | Estradiol | Estradiol significantly increased DPPIV activities of the 8 Prevotella strains in a strain- and dose-dependent manner. Both *P. aurantiaca* strains showed the highest DPPIV enzyme activities but were the poorest biofilm formers. | Estradiol regulates the DPPIV enzyme activity of *P. intermedia, P. nigrescens, P. pallens*, and *P. aurantiaca* strains differently. |
| (Larsen et al., 2006) | *C. albicans* strains | Estradiol, Progesterone | Estradiol induces the expression of *CDR1* and this correlates with growth. Progesterone also induces the expression of *CDR1*. | Estrogen induces *CDR1* expression at non-physiological doses (too high), whereas progesterone stimulates *CDR1* at physiological concentrations.  The virulence effects of *C. albicans in vivo* are likely induced by progesterone instead of oestradiol. |
| (Krishnamurthy et al., 1998) | *C. albicans* strains | β-Estradiol, Progesterone | β-Estradiol and progesterone enhance *CDR1* expression. | A steroid-receptor cascade linked to multidrug resistance of *C. albicans* is hypothesized, based on the responsiveness to human steroids present in the promoter region of *CDR1.* |

| Reference | Microbial species | Substrate | Results | Main conclusions |
| --- | --- | --- | --- | --- |
| (Alves et al., 2014) | *C. albicans* strains | Progesterone | Progesterone reduces capacity of *C. albicans* to form biofilms, colonise and invade epithelial cells. It also decreases the expression of *BCR1* and *HWP1.* | The observed effects of progesterone on *C. albicans* highlight the important role of this hormone in the progression of epithelial cell-invasion. This could explain susceptibility to vulvovaginal candidiasis at different stages of the menstrual cycle. |

**Table 2.** Summary of the available studies that have investigated the relation between fluctuating hormone levels and changes in the oral microbiota (not included in (Kumar, 2013))

| Reference | Study type | Population | Clinical measurements | Hormonal measurements | Microbiological samples and measurements | Results and main conclusions |
| --- | --- | --- | --- | --- | --- | --- |
| (Tarkkila et al., 2010) | Longitudinal | 106 healthy HRT-users and 55 healthy non-users (cohorts aged 50, 52, 54, 56 and 58) examined at baseline and after 2 years. | CPITN and panoramic radiographs | No | SGP collections from deepest pockets in each quadrant (pooled) using curettes. Analysis of *A. actinomycetemcomitans, P. gingivalis, T. forsythia., T. denticola, P. intermedia* and *P. nigrescens* using qPCR. | HRT users showed significantly fewer positive cases of *P. intermedia* and *T. forsythia* after 2 years compared to the non-users. The use of HRT did not correlate to periodontal health status. |
| (Emmatty et al., 2013) | Cross-sectional | 30 systemically healthy pregnant women equally divided into 1^st^, 2^nd^ and 3^rd^ trimester of pregnancy and 10 age matched non-pregnant controls (25-30) | PI, GI, PD | No | SGP collected using paper points.  Isolation of *F. nucleatum, A. actinomycetemcomitans, P. micra, P. intermedia* and *P. gingivalis* by inoculation into selective and non-selective media and identified by gram staining and biochemical characteristics. | Increased GI scores and *P. intermedia* were observed during the 2^nd^ and 3^rd^ trimester of pregnancy compared to 1^st^ trimester and non-pregnant controls without changes in PI. |
| (Borgo et al., 2014) | Longitudinal | 9 healthy pregnant women (18-35). Sampling and examination at 2^nd^ and 3^rd^ trimester.  9 healthy age-matched non-pregnant women as control. | VPI, GBI, PD, CAL, BOP | No | SGP collected using paper points and quantitative detection of A. a*ctinomycetemcomitans, F. nucleatum, P. gingivalis and P. intermedia* by qPCR. | Increase of gingival inflammation between the 2^nd^ and 3^rd^ trimester of pregnancy. Significant presence of *A. actinomycetemcomitans* among pregnant women at 2^nd^ and 3^rd^ trimester. *F. nucleatum* and *P. intermedia* were observed in high levels in the non-pregnant group. Pregnant women are more susceptible to gingivitis. |
| (Brusca et al., 2014) | Cross-sectional | 92 systemically healthy bodybuilders divided into anabolic androgenic steroid (AAS) users (N=42) and non-users (N=50) (19-40) | GI, PI, PD, CAL, number of teeth, radiographical examination | No | SGP (pooled) collected using paper points from the deepest pockets in each quadrant.  Identification of bacterial species (*P. gingivalis, A. actinomycetemcomitans* and *P. intermedia*) and *Candida* species was performed by means of morphological  and biochemical properties. | AAS users presented significantly  higher proportions of *A. actinomycetemcomitans, P. gingivalis, P. intermedia,* and *Candida* species compared to controls as well as higher GI and CAL, indicating that AAS use could increase the risk of severe periodontitis. |
| (Machado et al., 2016) | Longitudinal | 31 healthy pregnant women evaluated in the 2^nd^ trimester, 48 hours and 8 weeks after delivery (24-32) | BOP, PC, PD, CAL | No | Supra and SGP were collected Analysis using FISH to identify *A. actinomycetemcomitans, T. forsythia, C. rectus, P. gingivalis, T. denticola, F. nucleatum, P. intermedia* and *P. nigrescens.* | No significant differences were found in total bacterial counts between pregnancy and post-partum. There was a significant decrease of *P. nigrescens.*  High dropout rate. |
| (Paropkari et al., 2016) | Cross- sectional | 22 pregnant (1-24 weeks) and 22 non-pregnant systemically and periodontally healthy women (smokers and non-smokers) and 44 controls (18-35) | No  Only periodontally healthy women were included | No | SGP using paper strips was collected and analysed by means of 16S-pyrotag sequencing | Significant clustering of study subjects based on pregnancy and smoking status. Subgingival microbiomes of pregnant women and smokers showed commensal depletion. Smoking apparently promotes the growth of anaerobes while pregnancy promotes the growth of gram-positive and negative facultatives. |
| (Fujiwara et al., 2017) | Longitudinal | 132 healthy pregnant women assessed at 3 timepoints during pregnancy (22-35) and 51 healthy non-pregnant women as control (20-36) | No | No | Unstimulated saliva and SGP using paper points were collected. Seven microbial species were analysed by culturing and polymerase chain reaction (PCR) | Higher microbial counts during pregnancy.  Higher incidence of *P. gingivalis* and *A. actinomycetemcomitans* during first and second trimester.  Higher incidence *Candida* during second and third trimester. |
| (Lin et al., 2018) | Longitudinal | 11 systemically healthy pregnant women assessed on each pregnancy trimester and after delivery (24-29) and 7 non-pregnant controls (25-26) | PLI, GI, SBI | Salivary estradiol and progesterone | Supragingival plaque samples were collected and analysed by means of 16S rRNA sequencing | Diversity was significantly higher in the 3^rd^ trimester pregnancy group compared to the controls.  A distinct clustering according to gestational status was observed.  It can be suggested that ecological shifts take place during pregnancy promoting dysbiosis. |
| (Balan et al., 2018) | Cross-sectional | 10 healthy Chinese women on each pregnancy trimester and 10 in the post-partum period (26-37) | GBI, PI | No | Unstimulated saliva and SGP using paper cones were collected and analysed by means of 16S rRNA sequencing | Oral microbiome remained stable during pregnancy. There was a significant reduction of pathogenic species (*Veillonella parvula, Prevotella species* and *Actinobaculum species*) in the post-partum period compared with the three trimesters of pregnancy. |
| (Massoni et al., 2019) | Cross-sectional | 16 pregnant women on their 1^st^ trimester of pregnancy, 21 on the 2^nd^ trimester, 15 on the 3^rd^ trimester and 15 non-pregnant controls (18-35) | CAL, PD, PI, GI | Serum estradiol and progesterone | SGP collections using paper cones. The absolute identification and quantification of *A. actinomycetemcomitans*, *P. gingivalis, T. forsythia* and *S. oralis* by qPCR | Clinical diagnosis positively correlated to total bacterial count during pregnancy*.* *T. forsythia* was more frequently observed during the 1^st^ trimester and was also associated to an increase in gingivitis cases in pregnant women. *P.gingivalis* positively correlated with progesterone levels. |
| (Akcalı et al., 2014) | Cross-sectional | 45 women with PCOS healthy periodontium, 35  with PCOS and gingivitis, 25 systemically and periodontally  healthy, and 20 systemically healthy with gingivitis (25-32) | BOP, PD, PI | No | Unstimulated saliva was collected and analysed by means of qPCR. | Bacterial counts in participants with PCOS and gingivitis were significantly higher for *F. nucleatum, P. gingivalis, P. intermedia,* *S. oralis* and *T. forsythia.* Serum antibody levels to *P. gingivalis,*  *P. intermedia* and *S. oralis* were elevated in the presence of PCOS.  PCOS might influence the antigenic susceptibility to specific species. |
| (Lindheim et al., 2016) | Cross-sectional | 24 women with PCOS and 20 healthy controls (median 32) | No | Serum estrone, 17-estradiol, total testosterone, androstenedione, DHEA, DHEA-S and DHT | Unstimulated saliva was collected and analysed by means of 16S rRNA sequencing. | No significant differences in composition and diversity of the microbiome was observed between the groups.  Individuals with PCOS showed a decreased relative abundance of the phylum *Actinobacteria* |
| (Wendland et al., 2020) | Cross-sectional | 32 women with PCOS and 23 healthy controls (15-19) | GI, PD, PLI | Serum insulin, FSH, LH, total testosterone,  17-β-estradiol, DHEA-S,  and SHBG. | Unstimulated saliva was collected and analysed by means of real-time PCR. | No indication of a higher incidence of disease-associated bacteria. On the contrary, control group showed a higher incidence of bacteria from the red and orange complex. |
| (Bostanci et al., 2021) | Longitudinal | 43 women not using oral contraceptives, 41 women using combined oral contraception and 19 women using using levonegestrel intra-uterine system (22-28). All women were checked three times during their menstrual cycle (cycle day 1-3, 8-12, 18-22) | No | Plasma estradiol and progesterone | Stimulated saliva was collected and analysed using Next Generation Sequencing | Diversity of the microbiome was not significantly affected.  Salivary abundance of *Campylobacer,* *Haemophilus, Prevotella* and *Oribacterium* changed throughout the cycle.  The luteal phase presented a higher species-richness.  The menstrual cycle, contrary to contraceptive use, was responsible for greater variations in the metabolic pathways of the salivary microbiome. |

***Clinical parameters****. BOP= bleeding on probing; CAL= clinical attachment level; CPITN= community periodontal index of treatment needs; DHEA= dehydroepiandrosterone; DHEA-S= dehydroepiandrosterone sulfate; DHT= dihydrotes-tosterone; FSH= follicle-stimulating hormone; GBI= gingival bleeding index; GCF= gingival crevicular fluid; GI= gingival index; LH= luteinizing hormone; PC= presence of calculus; PD= probing depth; PBI= papillary bleeding index; PI= plaque index; PLI= plaque index; SBI= sulcus bleeding index; SGP= sub-gingival plaque; SHBG= sex hormone–binding globulin; PI= visible plaque index.*
